# Foreground Detection with Deeply Learned Multi-Scale Spatial-Temporal Features

**DOI:** 10.3390/s18124269

**Published:** 2018-12-04

**Authors:** Yao Wang, Zujun Yu, Liqiang Zhu

**Affiliations:** 1 School of Mechanical, Electronic and Control Engineering, Beijing Jiaotong University, Beijing 100044, China; wangyao@bjtu.edu.cn (Y.W.); zjyu@bjtu.edu.cn (Z.Y.); 2 Key Laboratory of Vehicle Advanced Manufacturing, Measuring and Control Technology (Beijing Jiaotong University), Ministry of Education, Beijing 100044, China

**Keywords:** fully convolutional networks, 3D convolutional networks, foreground detection, background modeling, deep learning, deep neural networks

## Abstract

Foreground detection, which extracts moving objects from videos, is an important and fundamental problem of video analysis. Classic methods often build background models based on some hand-craft features. Recent deep neural network (DNN) based methods can learn more effective image features by training, but most of them do not use temporal feature or use simple hand-craft temporal features. In this paper, we propose a new dual multi-scale 3D fully-convolutional neural network for foreground detection problems. It uses an encoder–decoder structure to establish a mapping from image sequences to pixel-wise classification results. We also propose a two-stage training procedure, which trains the encoder and decoder separately to improve the training results. With multi-scale architecture, the network can learning deep and hierarchical multi-scale features in both spatial and temporal domains, which is proved to have good invariance for both spatial and temporal scales. We used the CDnet dataset, which is currently the largest foreground detection dataset, to evaluate our method. The experiment results show that the proposed method achieves state-of-the-art results in most test scenes, comparing to current DNN based methods.

## 1. Introduction

Foreground detection, which extracts moving objects from the background of videos, is often considered as a key step in many video analysis tasks, e.g., intelligent surveillance, action recognition, object tracking, etc. It also has important applications in intelligent transportation systems [1,2]. Several excellent surveys address overall reviews of both traditional and recent methods in this field [3,4,5]. Conventional foreground detection methods normally build a model of the background of a video by various background modeling methods, and the foreground is then detected by finding the regions in input frames that are incompatible with the background model. Therefore, foreground detection is sometimes referred as background subtraction, whose performance largely dependents on the quality of the background model. One of the main difficulties of background modeling is the uncertainty and the randomness of the background. That is, the background changes in real surveillance situations due to many factors, e.g., illumination changes, moving background objects (water waves, waving trees, etc.), shadows, vibrations of cameras, etc., even when the videos are shot by stationary cameras.

Classic background models, e.g., Mixture of Gaussian (MoG) [6] and Kernel Density Estimation (KDE) [7], use statistics of color or intensity of individual background pixels to represent the background. These pixel-wise statistic methods can deal with repetitive background motions, but will have poor results when the background is highly dynamic since they ignore the spatial dependencies of neighboring pixels.

To improve the results for videos with highly dynamic background, researchers have proposed various regional models that explicitly take the dependencies of neighboring pixels into account. They are more robust with illumination changes and the dynamic background caused by a variety of nominal motions. The typical techniques include computing local features to integrate the information of local neighborhoods [8,9,10,11], or using Markov Random Fields (MRF) to explicitly model the dependencies of the neighborhood by introducing a prior probability to form the Maximum A Posteriori (MAP) framework [12].

However, all these methods use hand-craft features designed for particular problems, which are not necessarily ideal for all situations. Recently, Deep Neural Networks (DNN) are proven to be able to learn image features effectively, and have achieved great success in many tasks in computer vision, such as image recognition, object detection, etc. [13,14,15,16,17]. Especially, convolutional nerual network s (CNN) [18] are widely used for processing image and video information. Therefore, it is natural to apply DNN to foreground detection problem. Numerous CNN based foreground detection methods [19,20,21,22,23,24,25,26] have been proposed, and demonstrate significant improved results comparing with traditional methods. However, most only use spatial features. Although some works take the temporal information into account, they are either too sophisticated to detect efficiently or too simple to learn the temporal information effectively.

In this paper, we propose a novel background subtraction approach with dual multi-scale 3D fully convolutional network (DMFC3D), which is based on the 3D convolutional (C3D) network [27] originally designed for action recognition. The proposed method uses an encoder–decoder network. The encoder is first pre-trained using a massive action recognition dataset, and then the decoder is trained using foreground detection dataset with ground truth. Finally, the network is fine-tuned for each scene with a small set of pre-annotated image from the same video. The trained network can extract the spatial-temporal features from the input image sequence, and detect the foreground segmentation. Our main contributions include:

- We proposed a novel dual multi-scale 3D fully convolutional network structure for foreground detection. The network can learn deep and hierarchical multi-scale features in both spatial and temporal domains effectively, therefore has good performance for foreground detection of complex scenes.
- Our network demonstrates state-of-the-art performance comparing with other DNN based foreground detection methods, according to the experimental results on public datasets.
- The proposed DMFC3D network is a novel framework that establishes a mapping from image sequence inputs to pixel-wise classification results. It can be used for similar tasks that can benefit from effective multi-scale spatial-temporal features.

The remainder of this paper is organized as follows. In Section 2, we review the related works on both classic background modeling and recent methods using CNNs. Section 3 explains the proposed DMFC3D network structure for foreground segmentation. Section 4 introduces and discusses the experimental results. The last section summarizes our conclusions.

## 2. Related Works

### 2.1. Classic Background Models

Background modeling and foreground detection have been an active research area in the computer vision community for over two decades. Many methods have been proposed to overcome various difficulties. These methods can be approximately divided into three categories: pixel-wise models, regional models and global models. Pixel-wise methods model the changes of the color or intensity of individual pixels. Typical models include the Mixture of Gaussian (MoG) [6], the Kernel Density Estimation (KDE) [7] and various methods based on these two models [8,9,28,29]. The MoG is a parametric statistical model, which uses a linear combination of Gaussian models to describe the color distribution of each pixel in the image sequence. As a parametric model, the need to specify the key parameter (number of Gaussian models) is a main limitation of this approach. The KDE, on the other hand, is a non-parametric model and is driven by data. The estimator stores the history samples of each pixel, and then uses a kernel function to estimate the compatibility of the new observed data with the stored data. The pixel-wise statistical models have good results, when repetitive background motions are encountered. However, these models ignore the spatial dependencies of neighboring pixels, the foreground detected by these models usually have noise like false detections in highly dynamic background regions.

Regional models explicitly take the dependencies of neighboring pixels into account, so they are more robust with illumination changes and the dynamic background caused by a variety of nominal motion. Many authors proposed various local features to integrate the information of local neighborhoods. Liao et al. [8] introduced a scale invariant local ternary pattern (SILTP), which is motivated by the Local Binary Pattern (LBP) [30], and used a modified KDE to model the probability distribution of SILTP. Yoshinaga et al. [9] proposed a statistical local feature, which is obtained by applying a MoG to a local feature representing the difference of the target pixel and its neighboring pixels, to cope with illumination changes and periodical texture changes. Another category of widely used regional models are based on Markov Random Fields (MRF). MRFs explicitly model the dependencies of the neighborhood by introducing a prior probability to form the Maximum A Posteriori (MAP) framework. Sheikn and Shah [12] proposed a MAP-MRF framework, which uses both background and foreground models, and the MAP optimization of the framework is performed using the Graph-Cut algorithm. Generally, regional models have better result than pixel-wise models, but the computation load of regional models is so large that they are impossible to be applied in real-time systems.

Global methods [31,32], which use frame-level information to model the background of videos, is more robust to the global background charges. Oliver et al. [31] used eigenspace to model the background, and principal component analysis (PCA) to reduce the dimensionality of the image space. Their background model is represented by the eigenvectors corresponding to the *M* largest eigenvalues of the covariance matrix of training images. This approach is robust with global changes. Since the dimensions of the image are huge, the computation of eigenvalues and eigenvectors is difficult and requires considerable time.

Besides the approaches mentioned above, some neural network based foreground detection methods, which are closely related to the deep learning based methods, are also proposed [33,34,35,36,37,38]. Typical methods include Self-organizing Background Subtraction (SOBS) [33,34], and Background Neural Networks (BNN) [38]. SOBS use a self-organizing mapping to reduce the dimensions of the input image patterns while preserving the neighborhood topology, and can model the dynamic background effectively. The BNN is an unsupervised Bayesian classifier implemented by an artificial neural network to classify the pixel into foreground or background. However, these methods cannot deal with all the difficulties in complex scenarios.

### 2.2. Deep Learning Based Methods

Recently, with the great success of deep learning in many computer vision areas, many researchers proposed numerous foreground detection methods based on convolutional neural networks (CNN) [19,20,22,23,26,39]. One of the problems of applying CNN to foreground detection is that the CNN is originally designed for image classification, but foreground extraction needs pixel-wise result. One approach to overcome this problem is dividing image into fix-sized blocks. To our best knowledge, the possibility of using deep convolutional neural networks for background subtraction was first investigated by Braham and Van Droogenbroeck [26]. They used a network similar to LaNet-5 [18] to learn the background subtraction. For each pixel, the neighborhood of the pixel, along with the corresponding background, are inputed into the network, and the network output the classification result of whether that pixel belongs to background or foreground. Babaee et al. [20] used a similar paradigm, which inputs a block of image and the corresponding background block into a CNN, but outputs the probabilities of each pixel in the block belonging to the foreground. Another approach is using fully convolutional network (FCN) to achieve pixel-wise classification results. Cinelli et al. [22] proposed a FCN network based on ResNet [13] to extract a feature map from a pair of input frame and background, and used a reconstruction network to obtain the pixel-wise segmentation. In [23], the authors proposed a two-stage CNN: the first stage uses an encoder–decoder network to reconstruct backgrounds from input frames, while the second stage also use a FCN to get the pixel-wise segmentation from input frames and the reconstructed background. Zeng [19] introduced a multi-scale FCN, which uses a pre-trained VGG-16 network [15] as the basis, to learn multi-scale feature from input frames, and de-convolutional operation is used to obtain the pixel-wise classification results.

Beside the supervised CNN based methods, some unsupervised foreground detection approaches using Generative Adversarial Networks (GAN) are also proposed [40,41]. Sultana [40] used a GAN based context prediction method to reconstruct background from input image sequences. The foreground is then detected by subtracting images from the generated background. Bakkay et al. [41] proposed a background subtraction approach based on conditional Generative Adversarial Networks (GAN), where a generator learns the mapping from input to foreground detection result, and a discriminator learns to distinguish real or fake foreground. The generator uses 2D encoder–decoder architecture, therefore only spatial features are extracted. Unsupervised methods can be used in scenarios where ground truth is unavailable, but their performance is still not competitive with supervised methods.

However, most of the present deep learning based foreground detection methods use 2D convolutional network, therefore ignoring the temporal features of image sequences. Only a few works propose utilizing the temporal feature [21,24,42,43]. Yang et al. [21] proposed a FCN that use a clip of image sequence as input. All the frames are stacked together to form a multi-channel image, which is then processed using the 2D FCN. Chen et al. [24] used LSTM [44], which is placed after an encoder–decoder network, to learn the temporal features from the spatial features extracted by a FCN network. Hu et al. [43] also used LSTM, but the features are extracted by a 3D Atrous convolutional network, which can capture both spatial and temporal feature. Sakkos et al. [42] also proposed a 3D CNN architecture for background subtraction, and therefore can access to both spatial and temporal features. However, these methods do not use multi-scale approaches or only adopt multi-scale spatial information.

## 3. Proposed Method

Convolutional neural networks widely used in computer vision tasks are normally composed of convolution layers, pooling layers and fully-connected layers. Current fully-connected layers require the input and output to have fixed size. This limitation is a major problem for applying CNN to foreground detection, which needs pixel-wise classification results for input images with arbitrary size.

One simple approach is dividing the input image into small blocks with fixed size, but a foreground object may be divided into different blocks and hence the network cannot extract the feature of the whole object. Another solution is removing the fully-connected layers in CNNs, the resulting networks are called fully-convolutional network (FCN). FCN proposed in previous works use 2D convolutional operations, therefore can only learn spatial features.

In this work, we propose a multi-scale 3D FCN architecture, which uses 3D convolutional operations, can learn multi-scale features in both spatial and temporal domain. Multi-scale features are proven to be beneficial for many computer vision tasks, e.g., image recognition and semantic segmentation [45]. It is also shown that multi-scale spatial features can improve the results for foreground detection [19], because the object in the image can be different in size. In this paper, We show that multi-scale temporal features are also important for background detection, since different objects in the video can move at different speeds.

In this section, we introduce the detail of our proposed network for foreground detection.

### 3.1. Network Structure

**Figure 1:**
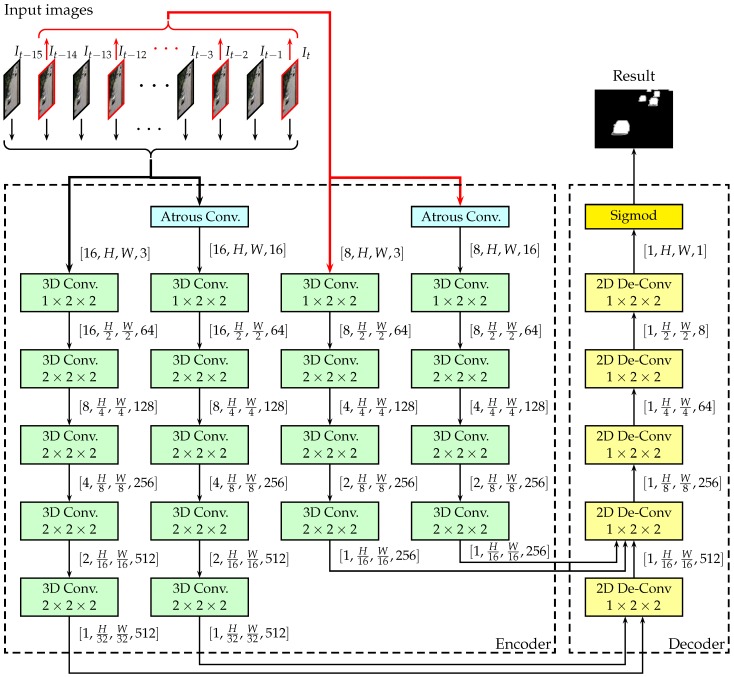
The architecture of the proposed network. (The downsampling rate or the upsampling rate are shown in each layer. The dimensions of the tensors are shown beside corresponding arrows.)

The architecture of our DMFC3D network is shown in Figure 1. The network proposed here uses an encoder–decoder structure. The input of the network is a clip of images with 16 continuous frames, including the current frame and 15 previous frames. The encoder extracts multi-scale spatial-temporal features (two spatial scale and two temporal scale) from the input sequences, and the decoder merge the features to reconstruct the pixel-wise detection result, which is the probability of each pixel belonging to the foreground, for the current image. The probability can be thresholded to form the binary classification result. Therefore, the network establishes a mapping from a clip of image sequence to the pixel-wise classification results.

The neural network is first trained by a two-stage training procedure, the encoder is first trained using a publicly available action recognition dataset, and then the decoder is trained by the foreground detection dataset. The trained network then can be used in the detection stage; when an image sequence is inputed, the network will give the foreground detection result of the inputed image.

The detail descriptions of encoder and decoder are given in the following subsections.

### 3.2. Encoder

The encoder extracts features from the input image sequence. The basic structure of the encoder is similar to the original C3D network [27], except we removed the fully-connected layers of C3D, and we used four paths to learn two different scales of temporal features and two scales spatial features. Each path has 4–5 levels of 3D convolutional layers, as shown in Figure 1, to learn deep spatial-temporal features of a particular scale.

#### 3.2.1. 3D Convolutional Layers

**Figure 2:**
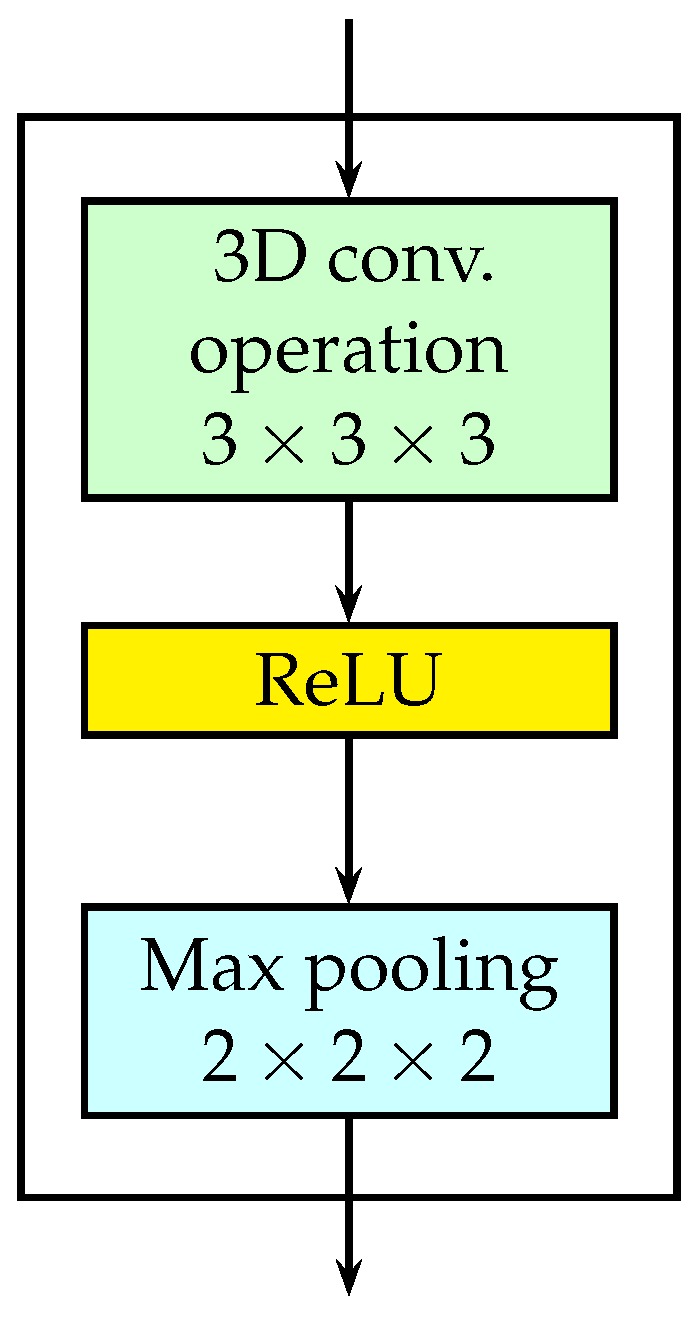
The detailed structure of a 3D convolutional layer.

The structure of a typical 3D convolutional layer is shown in Figure 2. It is composed of three sublayers: a 3D convolution operation sublayer, a nonlinear sublayer and a pooling sublayer.

A sequence of multi-channel images can be represented as a 4D tensor V. A pixel in V can be indexed as $V_{t,i,j,k}$, where the subscripts are listed in the order [T,H,W,C], and *T*, *H*, *W*, and *C* are the dimensions of time, height, width and channel, respectively.

The 2D convolution operation can be represented as Equation (1)

(1) $$Z_{i,j,k}=\sum_{m,n,c}V_{i+m-1,j+n-1,c}K_{m,n,c,k}$$

where V is the input tensor, Z is the output tensor, and K is the kernel, which is a 4D tensor containing *k* filters. Each filter performs the multi-channel 2D convolution with the inputs, the result of each convolution are considered as a channel of the output image. Here, the 2D convolutional operation for multi-channel images can be viewed as conducting convolution for each channel individually, and then computing the weighted sum of the results for each channel.

In constant, the 3D convolution operation, as represented in Equation (2), conducts convolution in three dimensions.

(2) $$Z_{t,i,j,k}=\sum_{d,m,n,c}V_{t+d-1,i+m-1,j+n-1,c}K_{d,m,n,c,k}$$

Therefore, 3D convolution layers in the DNN can learn parameters in the temporal dimension to represent the temporal feature effectively. Compared with the approaches that concatenate image sequence along the channel dimension and use the 2D convolutions, 3D convolutions can separate the temporal and spatial features instead of confounding them together.

As shown in Figure 2, a nonlinear sublayer is placed after the 3D convolution. We used ReLU (max(0,x)) as the nonlinear sublayer in the encoder. Finally, a pooling layer is added after the ReLU layer. The pooling operation outputs a summary statistic of the nearby inputs. We used max pooling, which reports the maximum output within a rectangular neighborhood, in the encoder. More specifically, the 3D max pooling is used to make the representation approximately invariant to small translation of the input in both temporal and spatial dimensions.

In the encoder, the kernel size of the 3D convolution is fixed to be 3×3×3 with stride 1. The downsampling rate of the pooling is shown the corresponding boxes in Figure 1.

#### 3.2.2. Multi-Scale Spatial Features

To get different spatial scales, we used Atrous convolution [45], which is a powerful tool in dense reduction tasks to create different scales without reducing the resolution. For each temporal scale in the DMFC3D, we split each temporal path into two spatial scales; in one scale, we used an Atrous convolution layer, which use a 3×3 kernel with rate 2.

#### 3.2.3. Multi-Scale Temporal Features

We also introduced a multi-scale temporal feature approach to improve the invariance to the different moving speed of foreground objects. As shown in Figure 1, we used a simple but effective sampling method to get two different scales in the time dimension. In the normal scale, we used a block of 16 continuous frames as input, which consist of the current frame and the 15 previous frames. If the current time is denoted as *t* and the current frame is denoted as $I_{t}$, the input images block is the set: $\left\{I_{t-15},I_{t-14},\ldots ,I_{t-1},I_{t}\right\}$. In 2× scale, the image sequence is sampled using 2× speed, so the input is the set: $\left\{I_{t-14},I_{t-12},\ldots ,I_{t-2},I_{t}\right\}$. Therefore, moving speed of foreground objects is doubled in this scale.

### 3.3. Decoder

The decoder is used to construct the pixel-wise foreground segmentation from the spatial-temporal features extracted by the encoder. The feature extracted by the encoder has low resolution but has many channels, with each channel representing a particular type of feature. Therefore, the decoder is required to merge these features and recover the resolution by a series of 2D de-convolutional layers, as shown in Figure 1. After the 2D de-convolutional layers, an output layer is added to represent the probability of pixels belonging to the foreground.

#### 3.3.1. 2D De-Convolutional Layer

**Figure 3:**
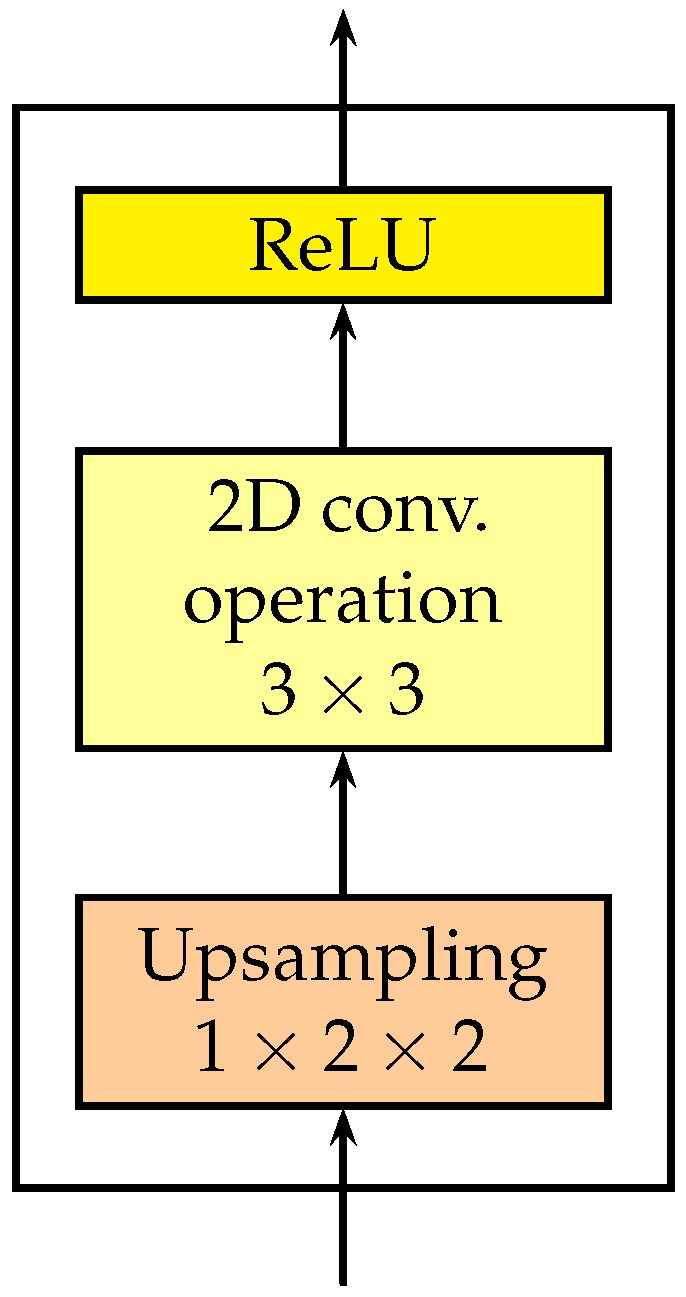
The detailed structure of a 2D de-convolutional layer.

The detailed structure of a 2D de-convolutional layer is shown in Figure 3. It is composed of three sublayers: an up-sampling sublayer, a 2D convolutional sublayer and a nonlinear sublayer.

After several layers of convolution and pooling in the encoder, the features have lower resolution compared to the input image. Up-sampling sublayers are used to recover the resolution. A simple up-sampling method is used. If the input of the up-sampling sublayer is denoted by V and the up-sampling scale is r×s, then the up-sampling operation can be represented by Equation (3).

(3) $$Z_{i,j,k}=V_{\left\lceil \frac{i}{r}\right\rceil ,\left\lceil \frac{j}{s}\right\rceil ,k}$$

where Z is the output. We only up-sample the spatial dimensions, so the time index is ignored here. The upsampling rate of the upsampling layers is fixed to be 1×2×2.

After the up-sampling sublayer, a 2D convolutional sublayer is added to merge the information in different feature channels. The kernel sizes of the 2D convolution are fixed to be 3×3.

#### 3.3.2. Output Layer

To represent probability distributions, we use the Sigmoid output layer, which is shown in Equation (4).

(4) $$\sigma \left(x\right)=\frac{1}{1+\exp(-x)}$$

The output range of Sigmoid layer is (0,1), representing the probability of a pixel belonging to the foreground.

### 3.4. Network Training

#### 3.4.1. Training Method

A two-stage training procedure with fine-tuning method is used to train the DMFC3D network. The encoder is first trained using publicly available massive action recognition datasets, then the decoder is trained by the foreground detection dataset, and finally the network is fine-tuned for each scenes. Training the DMFC3D network needs a lot of manually labeled training data, and the foreground detection datasets currently available are not large enough to train this network effectively.

In the first stage, we added a fully-connected layer after the encoder as a classifier. We trained the network using sports-1M dataset [46], which consists of 1 million videos annotated with 487 class and is much larger then any foreground detection dataset currently available. After training, the fully-connected layer was ignored, and the trained encoder could extract features from the input image sequences.

In the second stage, all parameters in the encoder trained in the first step were kept unchanged; only the parameters in the decoder were trained using the foreground detection dataset to learn how to recover pixel-wise foreground segmentation from the feature map provided by the encoder. We first used 50% of frames (along with their corresponding ground truth) from each scene as the training set, and the remaining data were used for evaluation. All the training data were combined together to train the decoder. Since the encoder was pre-trained, the training process for the decoder converged rapidly. Finally, the decoder was fine turned for each scene, using manually selected frames that contain foreground objects from the same scene in the training set. The amount of the selected frames was about 10% of the total number of the frames in the particular scene.

In both steps, we used the Adam algorithm [47] to train the network, with the learning rate set as 0.001. The kernels in the convolutional layers were initialized randomly with a normal distribution with standard deviation of 0.05 and the biases were initialized with a constant of 0.1.

As a supervised method, similar to other CNN based foreground detection techniques, ground truth is necessary to train the network, when applying our method to real scenes in practice. However, our two-stage training procedure makes it easier and quicker to train the network with limited number of ground truth. In practice, a few ground truth images may be obtain by conventional foreground detection methods or unsupervised approaches, in addition to some manually labeling works.

#### 3.4.2. Lost Function

In the second stage of the training, the weighted cross entropy was used as the lost function. Comparing with the normal cross entropy, a weight was added to emphasize the loss of a particular label, as defined in Equation (5)

(5) $$L\left(C,Z\right)=-\frac{1}{N}\sum_{n=1}^{N}\left(w\cdot C_{n}\log\left(Z_{n}\right)+\left(1-C_{n}\right)\log\left(1-Z_{n}\right)\right)$$

where *N* is the number of pixels, $C_{n}$ is the true label of *n*th pixel, and $Z_{n}$ is the predicted label of *n*th pixel, *w* is the weight.

Since only a small proportion of training images have foreground objects, and foreground objects are usually small, most pixels are background. Therefore, in the training data, there are many more background pixels than foreground pixels, i.e., the training data are highly imbalanced. The neural network trained by this imbalance training data will tend to classify foreground pixels to background. To reduce the impact of imbalance problem, we chose w>1 to amplify the loss for the mistake of classifying “1” to “0”.

## 4. Experiments

### 4.1. Dataset

**Figure 4:**
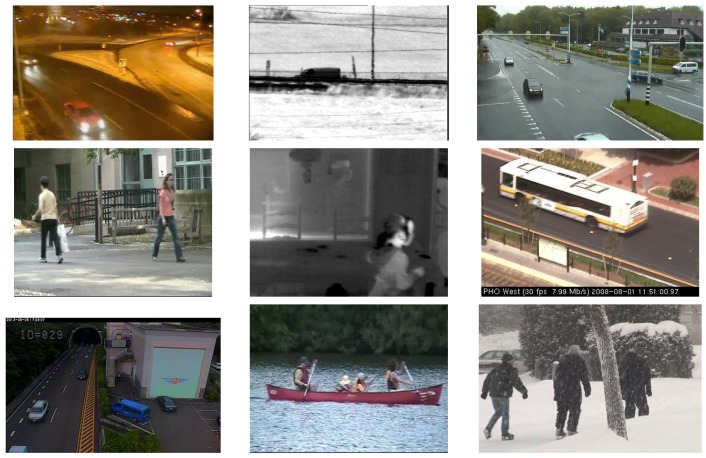
Example scenes in the CDnet 2014 dataset.

To train and test our network, we used the CDnet 2014 dataset [48], which is the largest dataset for background modeling and foreground detection. The dataset includes 53 videos in 11 categories. Each category, which has several videos of different scenes, focuses on a particular challenge for foreground detection, e.g., camera jitter, dynamic background, night videos, illumination change, etc. The dataset also provides a large number of hand-labeled pixel-wise ground truth for each video, which is useful for training and evaluating the network. The videos of most categories are captured by fixed cameras, except the PTZ category, of which the videos are captured by PTZ (Pan-Tilt-Zoom) cameras. All categories were used in the experiments. Some example scenes in the dataset are shown in Figure 4.

### 4.2. Evaluating Metric

Four raw statistics can be obtained by comparing the segmentation result with the ground truth: True positive (TP), False positive (FP), True negative (TN), and False negative (FN). Using these statistics, we can compute several evaluating metrics to compare the performance of different methods. Among them, F-measure (FM) is the most widely used evaluating metric for the performance of foreground detection task. F-measure can be computed by Equation (6).

(6) $$\mathrm{FM}=\frac{2\times \mathrm{precision}\times \mathrm{recall}}{\mathrm{precision}+\mathrm{recall}}$$

where precision=TP/(TP+FP) is the proportion of correctly detected foreground pixels in all the pixels detected as foreground; and recall=TP/(TP+FN) is the proportion of correctly detected foreground pixels in all the true foreground pixels. The range of FM is [0,1], and a higher FM indicates better performance.

FM does not consider TN; in the situation that the number of foreground pixels is small compared to the total number of pixels, the FM cannot evaluate the performance properly. Therefore, in addition to FM, PWC (percentage of wrong classifications) was adopted. The PWC metric takes TN into account, and is defined as:

(7) $$\mathrm{PWC}=\frac{100\times (\mathrm{FP}+\mathrm{FN})}{\mathrm{TP}+\mathrm{TN}+\mathrm{FP}+\mathrm{FN}}$$

PWC is the ratio of the number of misclassified pixels to the number of all pixels in the image. Its range is 0–100%, and a lower PWC indicates better performance.

### 4.3. Implementation Details

We used Tensorflow [49] to implement our network. The platform used in the experiments uses Intel i7-6850K CPU, and Nvidia GTX Titan Xp GPU.

Although the FC3D network proposed here does not require the size of input image to be fixed, we limited the size of the image to be not larger than 320×256 to fit the GPU memory. Because the encoder downsamples the image with scale of 32 and the decoder upsamples the feature map with the same scale, to get a detection result with the same size with the input image, the length and the height of the image need to be multiples of 32. In practice, we resized the image to change its length and height to the closest multiples of 32, and kept the size under the maximum size.

### 4.4. Results

We tested our method on the CDnet 2014 dataset, and compared with the current state-of-the-art methods including both CNN based methods and traditional methods.

#### 4.4.1. Results on CDnet 2014 Dataset

**Figure 5:**
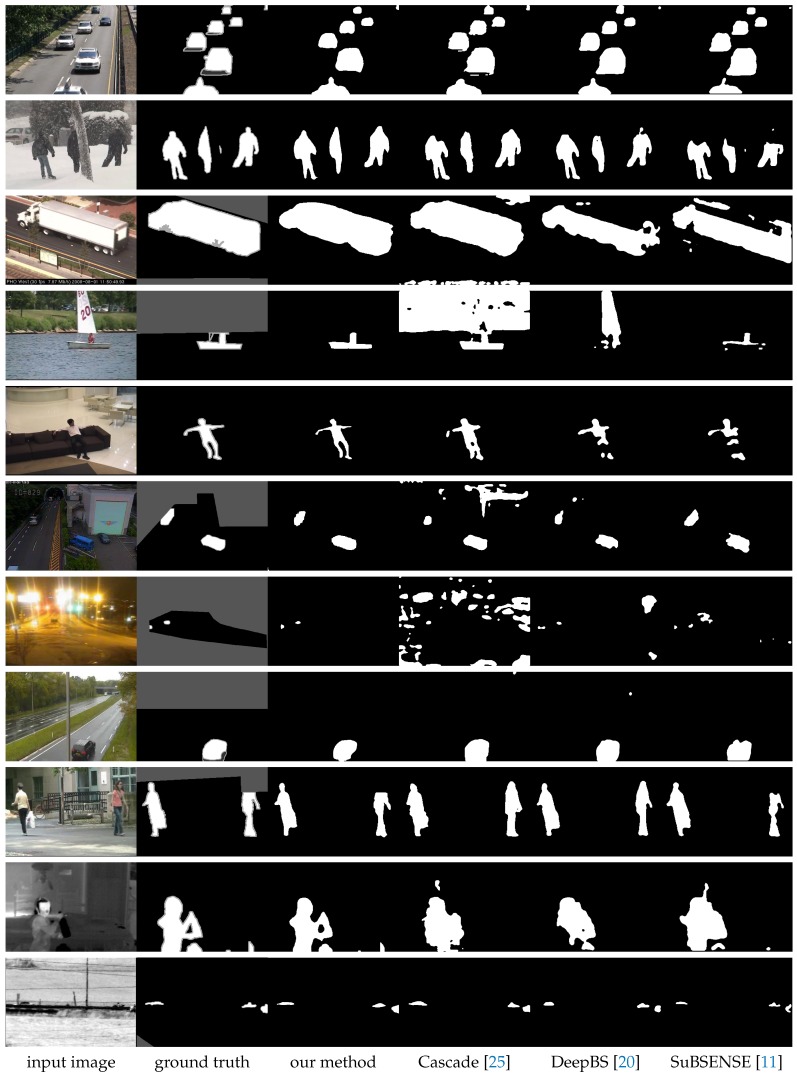
Results of CDnet 2014 dataset (From top to bottom: “baseline”, “badWeather”, “cameraJitter”, “dynamicBackground”, “intermittentObjectMotion”, “lowFramerate”, “nightVideo”, “PTZ”, “shadow”, “thermal”, and “turbulence”. The gray pixels in the ground truth indicate regions that are not interested).

Figure 5 shows the typical results of the DMFC3D as well as the results of comparing methods for CDnet 2014 dataset. Compared methods include two CNN based state-of-the-art methods, Cascade [25] and DeepBS [20], and a leading method of the traditional approaches, SuBSENSE [11]. For each category, one typical frame is selected and its input image, ground truth, segmentation results of different method are shown in the same row. In the results, white pixels represent foreground and black pixels stand for background.

In Figure 5, we can see that our DMFC3D network produces results very close to the ground truth for every scenes. Compared with the traditional approach, the CNN based methods show a significant improvement in the results, since they can learn better features of the input images from the training process. Among the three methods based on the CNN, our method produced obviously better results in the categories of “nightVideo”, “thermal” and “turbulence”, and, for other categories, the results of our DMFC3D network has similar results with the two other CNN based methods.

#### 4.4.2. Comparison with the State of the Arts

To compare the performance of our DMFC3D network with the state-of-the-art methods quantitatively, we computed the average F-measure and PWC of the results of DMFC3D and other comparing methods for each category in the CDnet 2014 dataset, as shown in Table 1 and Table 2, respectively.

The evaluation results provide more precise comparison. The DMFC3D has the best performances for most categories; especially in the categories of “nightVideo”, “thermal” and “turbulence”, the DMFC3D shows signification improvements compared with other methods (both CNN based and traditional approaches). This is because videos in these three categories have less notable spatial features: scenes are dark, and color information is lost. Therefore, the methods only based on the spatial features tend to have poor performance. In addition to the spatial features, the DMFC3D network can also learn the temporal features, which plays an important role for improving the performance when spatial features are ineffectual.

However, we found the temporal feature also induced some side effects. For example, the performance of the DMFC3D for the categories of “cameraJitter” and “PTZ” is slightly lower than the current leading method. The videos in these two categories have notable global motions caused by the camera movements. The global motions mislead the network during the training, and somehow prevent the network from learning effective temporal features.

In addition, we can see that for scenes in the “intermittentObjectMotion” category, our method produce relatively poor result comparing to other scene. That is because, in these scenes, temporal information of the foreground objects might be absent in some situations, for example: foreground object motion is intermittent or foreground objects are moving so fast that the same object is not present in two continuous frames. In these situations, the DMFC3D will perform similarly to other CNN based methods, since only the spatial features are available.

#### 4.4.3. Comparing to the Single Scale FC3D

To show the role of the multi-scale features, we implemented a single scale FC3D network as baseline to compare with the DMFC3D network. In the FC3D network, the encoder is simplified by only keeping the left most path in Figure 1, and the decoder is not changed.

The comparison the FM metric of DMFC3D and FC3D on CDnet2014 dataset is shown in Table 3. Generally, the DMFC3D network has better performance than the FC3D network for every category, although the improvement is minor for some categories. The improvement is caused by the multi-scale spatial-temporal features. For example, the DMFC3D has notable better results for the “lowFramerate” category, i.e., videos that are captured using very low frame rate. The objects in these videos move slowly, thus it is hard for the single scale FC3D network to learn effective temporal features of these slowly moving foreground objects. However, with multi-scale temporal features, DMFC3D shows good speed invariance to deal with low-speed foreground motion.

### 4.5. Running Time

Since the computation cost of 3D convolution is higher then 2D convolution, and a sequence of 16 images are needed for the detection of one frame. Therefore, the running time for foreground detection with the DMFC3D is longer may be a litter longer then other FCN based methods. With the platform and input size limitation specified in Section 4.3, the detection speed of our DMFC3D network is about 11–12 fps, which is still acceptable for many applications.

## 5. Conclusions

This paper proposed a novel deep neural network structure, referred to as dual multi-scale 3D fully-convolutional neural network (DMFC3D), for the foreground detection. The DMFC3D has an encoder–decoder structure, which extracts effective multi-scale spatial-temporal features from the image sequence input and inferences pixel-wise foreground object segmentation results from these features. The experimental results show that the performance of the DMFC3D foreground detection network exceed the current state of the art, benefited from its ability to learn deep and hierarchical spatial-temporal features effectively and its better invariance to the scale in both space and time dimensions. When applying our method to real scenes in practice, frames with ground truth from the same scene are required to fine-tune the network. However, only a small set of pre-annotated images is necessary and can be obtained by conventional unsupervised methods combined with some manual labeling. For future work, it is possible to consider using unsupervised methods to substitute some of the groundtruth masks.

The proposed network is also an effective framework to establish a map from image sequences to pixel-wise segmentation results. The experiments of the foreground detection has shown that temporal features and multi-scale features are helpful for improving the performance. It is possible to apply the DMFC3D network to other similar tasks that also need pixel-wise results from image sequences, e.g., crowd segmentation and abnormal event location, where features in the time dimension may have significant impacts for the classification results.

## Figures and Tables

**Table 1:** FM metric comparison of different foreground detection methods over all categories of the CDnet2014: baseline (BL), cameraJitter (CJ), badWeather (BW), dynamicBackgournd (DB), intermittentObjectMotion (IOM), lowFramerate (LF), nightVideo (NV), PTZ, shadow (SH), thermal (TH), and turbulence (TU).

Methods	BL	CJ	BW	DB	IOM	LF	NV	PTZ	SH	TH	TU	Average
DMFC3D (Ours)	**0.9950**	0.9744	**0.9703**	**0.9780**	**0.8835**	**0.9233**	**0.9696**	0.9287	**0.9893**	**0.9924**	**0.9773**	**0.9620**
Cascade [25]	0.9786	**0.9758**	0.9451	0.9658	0.8505	0.8804	0.8926	**0.9344**	0.9593	0.8958	0.9215	0.9273
DeepBS [20]	0.9580	0.8990	0.8647	0.8761	0.6097	0.5900	0.6359	0.3306	0.9304	0.7583	0.8993	0.7593
SuBSENSE [11]	0.9503	0.8152	0.8594	0.8177	0.6569	0.6594	0.4918	0.3894	0.8986	0.8171	0.8423	0.7453
PAWCS [50]	0.9397	0.8137	0.8059	0.8938	0.7764	0.6433	0.4171	0.4450	0.8934	0.8324	0.7667	0.7479

**Table 2:** PWC (%) comparison of different foreground detection methods over all categories of the CDnet2014: baseline (BL), cameraJitter (CJ), badWeather (BW), dynamicBackgournd (DB), intermittentObjectMotion (IOM), lowFramerate (LF), nightVideo (NV), PTZ, shadow (SH), thermal (TH), and turbulence (TU).

Methods	BL	CJ	BW	DB	IOM	LF	NV	PTZ	SH	TH	TU	Average
DMFC3D (Ours)	**0.0608**	0.2253	**0.0711**	0.0613	**1.1664**	**0.1201**	**0.2178**	0.2031	**0.0446**	**0.1301**	**0.0454**	**0.2133**
Cascade [25]	0.1405	**0.2105**	0.1910	**0.0522**	1.5416	0.1317	0.6116	**0.1221**	0.3500	1.0478	0.0584	0.4052
DeepBS [20]	0.2424	0.8994	0.3784	0.2067	4.1292	1.3564	2.5754	7.7228	0.7403	3.5773	0.0838	1.9920
SuBSENSE [11]	0.3574	1.6469	0.4527	0.4042	3.8349	0.9968	3.7717	3.8160	1.0120	2.0125	0.1527	1.6780
PAWCS [50]	0.4491	1.4220	0.5319	0.1917	2.3536	0.7258	3.3386	1.1162	1.0230	1.4018	0.6378	1.1993

**Table 3:** FM metric comparison of multi-scale FC3D and single-scale FC3D over CDnet2014.

Category	DMFC3D	FC3D
baseline	0.9950	0.9941
cameraJitter	0.9744	0.9651
badWeather	0.9703	0.9699
dynamicBackground	0.9780	0.9775
intermittentObjectMotion	0.8835	0.8779
lowFramerate	0.9233	0.8575
nightVideo	0.9696	0.9595
PTZ	0.9287	0.9240
shadow	0.9893	0.9881
thermal	0.9924	0.9902
turbulence	0.9773	0.9729
**Average**	0.9620	0.9524
